# Understanding Drivers of Phylogenetic Clustering in Molecular Epidemiological Studies of HIV

**DOI:** 10.1093/infdis/jiu563

**Published:** 2014-10-13

**Authors:** Simon D. W. Frost, Deenan Pillay

**Affiliations:** 1 Department of Veterinary Medicine and Institute of Public Health, University of Cambridge; 2 University College London, United Kingdom; 3 Africa Centre for Health and Population Studies, University of KwaZulu Natal, Durban, South Africa

**Keywords:** HIV, phylogenetics, epidemiology, clustering

**(See the major article by Poon et al on pages 926–35.)**

Despite declarations that the so-called end of AIDS is near, the global human immunodeficiency virus (HIV) epidemic continues to grow. This is the case within localized epidemics in the resource-rich world, as well as so-called generalized epidemics in the resource-limited setting. With the relative lack of success of widespread prevention approaches, focus has turned to the finer granularity of the epidemics [1]. Even within generalized epidemics in sub-Saharan Africa, evidence points to increasing heterogeneity in transmission [2]. More-precise determination of the characteristics of individuals continuing to spread the virus—for instance, whether their infection is undiagnosed, diagnosed and untreated, or diagnosed and treated—is needed to guide prevention to reduce transmission to manageable levels.

It is within this context that the use of viral genetic sequences can add important value to inference of transmission dynamics and inform targeted prevention strategies [3]. Sequence data are becoming increasingly used in epidemiological studies for a variety of pathogens, with recent recommendations for how such studies are to be reported [4]. Whereas the major focus for implementing molecular epidemiological approaches for HIV should be toward reducing the devastating epidemics in Africa and Asia, the vast majority of HIV sequence data derives from North America and Europe, mainly as a result of widespread HIV genotypic drug resistance testing. Nevertheless, sequence data from resource-rich settings represent an invaluable resource for developing methods that can be applied globally. This is particularly the case when sequence databases cover a significant fraction of individuals infected with HIV, as exemplified in countries such as the United Kingdom, Switzerland, and the Netherlands.

Use of phylogenetics to identify the likely source of specific transmission events is a well-trodden path in HIV research [5], particularly in relation to small, targeted epidemiological investigations. However, when applying such approaches to data sets sampled at a regional or national level, the sampling fraction is too low to detect significant numbers of direct transmissions [6]. Nevertheless, so-called clusters of highly similar viruses are often observed [7–9]. How these clusters are defined varies across studies, although all use a measure of distance between pairs of sequences, below which the sequences are deemed clustered. Some studies use genetic distances calculated directly from the sequence data, whereas others use distances calculated from a phylogenetic tree. Some phylogenetic studies use information on sampling times of the sequences, allowing clustering to be defined in terms of calendar time rather than in terms of the percentage divergence of the sequence. Furthermore, additional criteria may be used to classify clusters, such as the level of statistical support and the minimum size of a cluster. These differences in cluster definition make it difficult to compare the frequency of clustering in different studies, but it is apparent that such clustering is widespread and can even be detected between HIV sequences from different countries [10].

The demonstration of the clustering of a group of viruses by itself is not particularly useful, as clustering occurs even in a homogeneous population and can be driven by nonepidemiological factors, such as how individuals are sampled. However, when combined with other information about the individual, examination of clusters may reveal potential subepidemics and moves the focus to the characteristics of individuals within a cluster. Further insights can be gained by considering who an individual is clustered with. For example, coclustering of individuals with recent infection is a better indicator of the higher infectiousness of these individuals than clustering, which may simply reflect the limited time for the virus to diverge in these individuals [11].

In this issue of *The Journal of Infectious Diseases*, Poon et al present an analysis of HIV type 1 sequence data collected from British Columbia, Canada. This adds to the relatively few phylogenetic studies with a high level of coverage—approximately 50% of the HIV-positive population in British Columbia. Unlike other studies, multiple sequences per patient were considered when identifying clusters, which allowed Poon et al to identify more clustered individuals than if only the first available sequence for each individual was used. Although there may be a bias in those with multiple sequences, for the purposes of examining transmission, this bias may not be that important, as the availability of multiple sequences for an individual may reflect that the individual experienced a rebound in viral load during therapy and, hence, remained infectious. Building on similar work [12, 13], they use sequence data both to identify clustered individuals, as well as to define what they term a “phylogenetic neighborhood” for each individual. Characteristics of individuals within their phylogenetic neighborhood were related to whether individuals were clustered, allowing Poon et al to start to disentangle correlates of clustering and coclustering, while avoiding the attribution of the source of infection to any individual present in the sample.

Poon et al demonstrated that individuals were more likely to be clustered if the viral load in their phylogenetic neighborhood was higher. Studies of HIV-transmitting partners have demonstrated that the viral load in the infecting partner accounts for 20%–55% of the variation in viral load in the recipient partner [14]. As viral loads in 2 individuals are likely to show negligible correlation if separated by ≥5 transmissions, this implies that an individual is separated from individuals in their phylogenetic neighborhood by a limited number of intermediate transmissions.

Some information on likely seroconversion dates was available, although it was mainly determined from physician reports rather than on the basis of clear serological evidence. Consistent with other studies [15], individuals with recent HIV infection clustered together. When combined with information on sampling date, estimates of seroconversion dates also allowed changes in clustering over time to be investigated, demonstrating early establishment of clusters among people who inject drugs, followed by more-recent emergence of clusters among men who have sex with men (MSM). Although potentially confounded by changes in sampling patterns over time, this result is consistent with surveillance data over the past decade, demonstrating declining numbers of HIV infection diagnoses among people who inject drugs but sustained numbers of diagnoses among MSM [16]. This suggests that in other populations, the dynamics of clusters over time may provide insights into past transmission, even when classical epidemiological data are lacking [17]. The potential to map the impact of large-scale intervention strategies is currently being tested on epidemics in Africa, using full-length HIV sequences within the PANGEA-HIV consortium (available at: https://github.com/PangeaHIV).

Poon et al found that the presence of mutations that conferred resistance to nucleoside reverse transcriptase inhibitors (NRTIs) in an individual's phylogenetic neighborhood was associated with significantly less clustering. Although sustained transmission of drug resistance mutations has been found in other studies in the United Kingdom [18] and Switzerland [9], the underrepresentation of NRTI resistance is consistent with earlier reports arguing that the frequency of transmitted resistance is much lower than the number of potential transmitters of resistance at the population level [19, 20].

While clinical databases of HIV can offer epidemiological insights at little incremental cost, they are not without limitations. Individuals who are infected with HIV but have yet to receive a diagnosis are not sampled, and establishing the role of these individuals in ongoing transmission is essential to guide efforts to roll out testing in the community. Inclusion of samples obtained through anonymized surveillance programs, for example, may reveal additional clusters. In addition, only very basic information on demographic characteristics and risk factors is routinely collected during counseling and testing. Despite the many challenges in measuring contact networks [21], developing surveys that capture the dynamic and possibly network-dependent nature of risk behaviors may allow us to dissect the drivers of phylogenetic clustering in more detail.

A key question is whether phylogenetics adds significantly to careful epidemiological mapping of the epidemic. A limited number of studies to date suggest that sequence data can be informative about factors such as the stage of HIV infection when transmission occurs [22], as well as in identifying spatial structure [23]. In addition, such data have the potential to provide insights into superinfection and recombination. As detectable recombination at the population level takes place when the same individual is infected with multiple divergent viruses and then goes on to transmit a recombinant, better characterization of recombination may give further insights into groups with high transmission rates.

As HIV genetic data become even easier and cheaper to generate, the field has turned to the methodological and informatic challenges of making robust epidemiological inferences from next-generation sequence data [24]. Reliance on sequences alone, however, neglects uninfected individuals. While some attempts have been made to extract information about the underlying contact structure from patterns of phylogenetic clustering [25], more mechanistic phylodynamic models are needed that explicitly link the phylogeny of infected individuals in the sample to the population of infected and uninfected individuals. It is critical for large sequence data sets to be placed side by side with detailed clinical, epidemiological, and behavioral information, to maximize the potential of phylogenetic approaches.
